# An Update on Role of Ionizing Radiation to Enhance Proliferation and Differentiation of Normal Stem Cells via Activation of NRF2 Pathway: Review

**DOI:** 10.3390/antiox14080986

**Published:** 2025-08-11

**Authors:** Kave Moloudi, Siamak Haghdoost

**Affiliations:** 1 Laser Research Centre, Faculty of Health Sciences, Doornfontein Campus, University of Johannesburg, Johannesburg 2028, South Africa; moloudikave@gmail.com; 2 Laboratoire Aliments, Bioprocédés, Toxicologie Environnements (ABTE, UR4651), University of Caen, Normandy, Cedex 04, 14050 Caen, France; 3 Advanced Resource Center for HADrontherapy in Europe (ARCHADE), 14000 Caen, France; 4 Department of Molecular Biosciences, The Wenner-Gren Institute, Stockholm University, SE-10691 Stockholm, Sweden

**Keywords:** ionizing radiation, NRF2, differentiation, PI3K/Ak, osteogenesis, neurogenesis

## Abstract

Ionizing radiation (IR) as a stress inducer has a significant impact on various normal stem cells differentiation through activation of various signaling pathways. Low levels of oxidative stress of IR may preserve or even enhance cell differentiation. In response to IR, reactive oxygen species (ROS) can activate various signaling pathways that promote cell differentiation, notably through the involvement of nuclear factor erythroid 2–related factor 2 (NRF2). NRF2 interacts with multiple pathways, including Wnt/β-catenin (osteogenesis), PPARγ (adipogenesis), and BDNF/TrkB (neurogenesis). This response is dose-dependent: low doses of IR activate NRF2 and support differentiation, while high doses can overwhelm the antioxidant system, resulting in cell death. However, the quality of various types of IR, such as proton and carbon ion radiation, may have a varied impact on stem cells (SCs) differentiation compared to X-rays. Hence, activation of the NRF2 signaling pathway in SCs and cell differentiation depends on the level of stress and the quality and quantity of IR. This review is an update to explore how IR modulates SCs fate toward osteogenic, adipogenic, and neurogenic lineages through the NRF2 signaling pathway. We highlight mechanistic insights, dose-dependent effects, and therapeutic implications, bridging gaps between experimental models and clinical translation.

## 1. Introduction

Ionizing radiation (IR) as an oxidative stress factor can promote the differentiation of various stem cells such as adipose-derived stem cells (ADSCs) and mesenchymal stem cells (MSCs) [1]. IR via reactive oxygen species (ROS) such as hydroxyl radical and superoxide anion promotes osteogenic differentiation of ADSCs [2,3]. Moreover, the mitochondria as a source of ROS generation enhance the proangiogenic potential of ADSCs by increasing secretion of growth factors and protecting against oxidative stress-induced cell death [4]. H_2_O_2_ treatment also effectively induces chondrogenic differentiation of ADSCs, resulting in increased glycosaminoglycan content and upregulation of chondrogenic genes [5]. The balance between ROS and the cellular antioxidant system plays a critical role in regulating the microenvironment, as well as the properties and differentiation potential of ADSCs [6]. Notably, under oxidative stress conditions, reduced expression of the antioxidant regulator NRF2 enhances osteoblastic differentiation of ADSCs through an autophagy-dependent mechanism [6]. These findings highlight the complex interplay between oxidative stress and ADSC differentiation, suggesting potential therapeutic applications in tissue engineering and regenerative medicine.

Following IR, various signaling pathways work in concert to defend cells against damage, promote repair, and control differentiation. The DNA damage response (DDR), NRF2, PI3K/Akt pathway, Wnt/β-catenin pathway, NF-κB pathway, notch signaling, and hippo pathway each contribute to the regulation of stem cell behavior and tissue regeneration [1,7,8,9,10]. These pathways help orchestrate cellular decisions to repair radiation-induced damage, prevent cell death, and promote differentiation, ultimately aiding in the recovery of irradiated tissues [10]. Recent studies have explored the role of NRF2 in stem cell response to IR [1,11]. NRF2 activation has been shown to enhance hematopoietic stem progenitor cell (HSPC) function and mitigate radiation-induced myelosuppression and mortality in mice [12,13]. This protective effect is mediated through the activation of Notch signaling in HSPCs and increased Jagged1 expression in bone marrow stromal cells [13]. The NRF2-ARE (antioxidant response element) pathway interacts with other key mechanisms, including DNA repair (MRN complex) and inflammatory responses (HMGB1 and cytokines), to modify radiation damage [14]. Moreover, NRF2 activation enhances DNA repair, detoxifies superoxide, and maintains redox balance, thereby mitigating radiation-induced toxicity [11]. In normal human ADSC, NRF2 inhibition reduces stem cell differentiation capacity following radiation exposure, with the effect being radiation quality-dependent [1]. In addition to NRF2 signaling, Akt activation by IR leads to protection of cells from radiation-induced damage by promoting survival and repair processes. The PI3K/Akt pathway is involved in cell survival, metabolism, and proliferation [15]. Another important signaling pathway involved in differentiation is the NF-κB (nuclear factor kappa-light-chain-enhancer of activated B cells) pathway, which is a major regulator of immune responses, inflammation, and cell survival [16]. After radiation, NF-κB activation helps protect cells from radiation-induced apoptosis and promotes tissue repair. Therefore, in response to stress, the IκB kinase (IKK) complex activates NF-κB by phosphorylating IκB proteins, leading to the release and nuclear translocation of NF-κB dimers. NF-κB activates the transcription of genes involved in inflammation, survival (e.g., Bcl-2), and DNA repair [17,18].

However, the level of ROS induced by IR is a crucial factor in activating various signaling pathways to promote cell survival and cell differentiation and regulate antioxidant responses [11]. NRF2 activation depends on some critical factors, including dose (the amount of energy deposited by IR per unit mass of biological tissue, expressed in grays (Gy)) and dose rate (the rate at which radiation dose is delivered to a biological system, typically over time, typically expressed in Gy/min), cell type, level of antioxidants in the cell and environmental factors, and may occur after a delay, suggesting it acts as a second-tier antioxidant response system [19,20]. Hence, in this updated and critical review, we have been focused on NRF2 signaling after IR and its role in the differentiation and proliferation of various stem cells. Furthermore, the authors recommended some suggestions for future perspective.

## 2. IR Induces Activation of NRF2 Signaling Pathway via Oxidative Stress

Oxidative stress results from an imbalance between the production of ROS and the body’s ability to neutralize them, leading to an excess of ROS. ROS are highly reactive molecules, including free radicals such as superoxide anion (O_2_^•−^) and non-radical molecules such as hydrogen peroxide (H_2_O_2_) that can lead to cellular and molecular damage [21]. Various types of IR, such as alpha (α), beta (β), and gamma (γ)/X-rays, can have a variety of biochemical and physiological consequences, including oxidative stress. IR can directly ionize biomolecules such as DNA, proteins, and lipids, resulting in modifications of the biomolecules. IR can also indirectly modify biomolecules through its interaction with water molecules, leading to the radiolysis of water and the production of ROS, e.g., hydroxyl radicals (^•^OH), hydrogen radicals (H^•^), superoxide anion (O_2_^•−^), and hydrogen peroxide (H_2_O_2_), that are highly reactive and can damage various cellular components [22]. Consequently, this process can trigger a range of biochemical and cellular effects, including oxidative stress characterized by DNA damage, lipid peroxidation, loss of membrane integrity, and protein oxidation. Additionally, antioxidant defense systems (e.g., superoxide dismutase, catalase, and glutathione peroxidase) and non-enzymatic antioxidants (e.g., vitamins C and E) can scavenge ROS. Furthermore, DNA repair systems possess multiple repair pathways to correct oxidative DNA damage or single/double DNA strand breaks. In contrast, severe DNA damage can trigger apoptosis or necrosis as a protective mechanism to eliminate potentially damaged or dangerous cells [23,24].

However, IR is a key source of oxidative stress, and the dose and level of ROS production in tissues influence NRF2 activation [11,25]. Equal doses of various types of IR (photonic IR and particles) potentially cause varying levels of ROS and oxidative stress [1,26]. This variation arises due to differences in the physical properties of each type of radiation, such as their mass, linear transfer energy (LET), charge, and energy, which influence how they interact with biological tissues and induce oxidative stress [1]. For instance, low doses of carbon ions are more effective than X-rays in producing ROS in human fibroblasts [27]. Furthermore, the level of IR dose that induces oxidative stress is an important factor to consider, as stem cells are generally more sensitive to IR. Hence, NRF2 activation in response to oxidative stress typically begins at moderate radiation doses and reaches a plateau at very high doses [28]. In mesenchymal stem cells, low-dose IR (10 cGy) induces a modest transient increase in NRF2 protein levels (e.g., ~30% increase at 10 cGy), followed by a decline, whereas doses around 50 cGy give rise to sustained NRF2 elevation at 24 h post-irradiation [29]. Additionally, it is reported that in long-term hematopoietic stem cells, nuclear translocation of NRF2 occurs in only a small fraction (<10%) at very low doses (0.02 Gy) within the first hour, rising to ~60% at 2 h post-exposure; this suggests a dose- and time-dependent activation that peaks and then stabilizes [30].

However, during oxidative stress, the ROS produced oxidize cysteine residues on KEAP1, leading to conformational changes that impair its ability to bind and degrade NRF2 [31]. Consequently, NRF2 activation has a significant role in stem cell differentiation, proliferation, and huge enzymes [32,33].

### 2.1. Consequences and Antioxidant Effects of NRF2 Activation in Stem Cells

**Figure 1:**
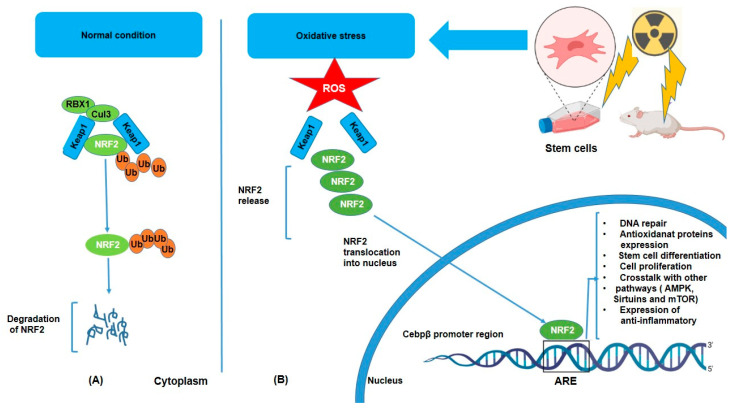
Degradation and activation of the NRF2 pathway under unstressed (**A**) and oxidative (**B**) conditions. NRF2 is released from KEAP1 and moves into the nucleus, where it stimulates the production of genes that code for antioxidant enzymes and other proteins. This system is crucial for redox equilibrium maintenance and cell defense against oxidative damage.

Under normal conditions, NRF2 is kept in the cytoplasm bound to a protein called KEAP1 (Kelch-like ECH-associated protein 1), which, in the absence of stress, binds to NRF2 and facilitates its ubiquitination (Ub) and degradation via the proteasomal pathway, but oxidative stress triggers its dissociation from KEAP1 and subsequent nuclear translocation [34] (Figure 1). KEAP1 has several cysteine residues that act as sensors for oxidative stress [35]. However, under stress conditions such as IR or inflammatory signals, NRF2 dissociates from KEAP1, translocates to the nucleus, and activates the transcription of target genes such as HO-1 (heme oxygenase-1), NQO1 (NAD(P)H dehydrogenase quinone 1), glutathione S-transferases (GSTs), superoxide dismutase (SOD), and glutathione peroxidases (GPx) [36,37]. These enzymes help neutralize ROS and reduce oxidative damage by breaking down or scavenging harmful molecules. Moreover, NRF2 activation helps preserve cellular integrity by promoting DNA repair mechanisms through the expression of DNA repair genes [38,39]. Furthermore, activation of NRF2 leads to enhancement of the production of lipid repair enzymes, which can restore lipid structures damaged by ROS [40]. Additionally, the anti-inflammatory effect of NRF2 is considerable in that chronic oxidative stress often triggers inflammation, a hallmark of various diseases. ROS can activate the NF-kB pathway, which promotes the production of pro-inflammatory cytokines (e.g., TNF-α, IL-6) and further exacerbates oxidative damage. Consequently, NRF2 can suppress inflammation by inhibiting NF-kB and downregulating the expression of pro-inflammatory cytokines and expression of anti-inflammatory molecules such as IL-10 [41,42,43]. Hence, in this way, NRF2 not only reduces oxidative stress but also prevents the harmful cycle of oxidative stress and inflammation. Another important role of NRF2 is crosstalk with other pathways such as AMPK (AMP-activated protein kinase), Sirtuins, and mTOR (mechanistic target of rapamycin) [44,45]. It means that NRF2 does not work in isolation, but that it interacts with other signaling pathways to fine-tune the cellular response to oxidative stress. For example, NRF2 with AMPK, a key regulator of energy metabolism and cellular stress response. AMPK can activate NRF2, and NRF2 activation can enhance AMPK signaling, promoting cellular survival under stress conditions. In addition, Sirtuins proteins, involved in the regulation of aging and metabolic health, can interact with NRF2 to modulate antioxidant responses and cellular longevity. NRF2 can influence mTOR activity, which in turn regulates cell growth and metabolism in response to oxidative stress. Together, all of these reactions following oxidative stress may demonstrate how NRF2 contributes to stem cell differentiation through interactions with PPARγ (adipogenesis) [46,47], BDNF/TrkB (neurogenesis) [48,49], and Wnt/β-catenin (osteogenesis) [46,47].

### 2.2. Role of Activation of NRF2 Pathway in Cell Differentiation After IR

#### 2.2.1. NRF2 Enhances Osteogenesis

**Figure 2:**
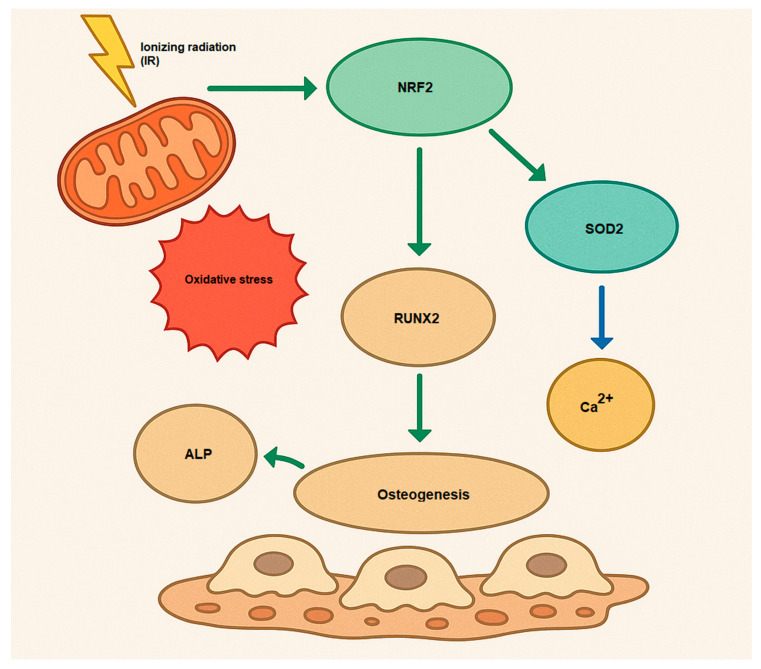
Osteogenesis mechanism via activation of NRF2 by IR (oxidative stress). Runx2, Runt-related transcription factor 2; ALP, alkaline phosphatase; Ca^2+^, Calcium; SOD2, superoxide dismutase 2, mitochondrial.

The NRF2 activation causes osteogenesis and bone homeostasis during a complex role. While NRF2 inhibition enhances differentiation in osteoclasts, the role of NRF2 in osteoblast differentiation is more nuanced, with both inhibition and activation potentially decreasing osteogenesis [50] (Figure 2). Hence, NRF2 signaling influences osteogenesis by regulating the expression of genes that promote osteoblast differentiation, such as Runx2 (Runt-related transcription factor 2) and Osterix (Sp7). These transcription factors are essential for initiating and maintaining osteoblastic differentiation [51,52]. Furthermore, NRF2 signaling also affects the mineralization process in osteoblasts. Osteoblasts secrete alkaline phosphatase (ALP) and collagen type I, both of which are essential for bone matrix formation and mineralization [53]. Additionally, NRF2 does not act alone; it interacts with several other signaling pathways involved in osteogenesis. For example, NRF2 can work synergistically with the Wnt/β-catenin signaling pathway, which is crucial for osteoblast differentiation and bone formation [54,55]. Moreover, NRF2 can modulate the TGF-β (Transforming Growth Factor-beta) signaling pathway, influencing both osteoblast differentiation and extracellular matrix production [56,57,58]. Understanding how to modulate this pathway may offer novel therapeutic approaches for bone diseases such as osteoporosis, and it highlights the importance of oxidative balance in skeletal health.

Here, some research has been summarized that IR caused stem cell differentiation in vitro and in vivo (Table 1). While some research has indicated that NRF2 loses its antioxidant capacity at high IR doses (>2 Gy) [59,60], it may be activated at low doses (<2 Gy) or fractionated doses, leading to osteogenesis and proliferation. Dominici et al., have reported that the 1.125 Gy of IR from ^137^Cs caused proliferation and differentiation of osteoblast cells via increasing of stromal-derived factor-1 level (SDF-1) [61]. Another study by Kim and colleagues shows that NRF2 activation enhances early hematopoietic reconstitution of bone marrow after 24 h of lethal IR exposure via upregulation of Notch signaling to improve HSC function in vivo. Their findings show that there is a significant increase in expression in KI67 (markers of proliferation), NRF2, Jagged 1 (JAG1), and KEAP1 [13]. Additionally, Xi et al., illustrated that t-BHQ (Solarbio) as an NRF2 activator enhanced differentiation of periodontal ligament stem cells (PDLSCs) osteogenic under cyclic mechanical stretch. T-BHQ may stimulate osteogenic differentiation both in vivo and in vitro, indicating a significant alternative for alveolar bone remodeling during orthodontic tooth movement through expression of HO-1 and NQO-1 genes [62]. Henry and co-workers evaluated the role of oxidative stress at high and low doses of IR (0.02–2.5 Gy) on HSCs, and their findings showed that IR at a 2.5 Gy dose caused cell death via DNA damage and oxidative stress. Furthermore, they noted that a low dose of IR (0.02 Gy) leads to self-renewal and differentiation of HSCs with a decrease in mitochondrial membrane potential and formation of 8-oxo-deoxyguanosine (8-oxo-dG) in DNA. They found that low-dose IR impairs HSPC function through ROS and p38/MAPK14, but not via classical DDR via ATM or p53 [63]. In contrast, Kook S-H et al., showed that IR suppressed the maturation and mineralization of osteoblasts through the activation of the NRF2/HO-1 pathway in MC3T3-E1 cells [64]. They used a linear accelerator radiotherapeutic machine (Mevaprimus, Siemens, Munich, Germany) in various doses (0–8 Gy at a rate of 1.5 Gy/min) for various incubation periods (0–28 days). Their findings indicated that IR with high doses (>2 Gy) inhibits osteoblast differentiation and mineralization with the decreased expression of bone-specific markers such as ALP, NO-1 and NRF2 in MC3T3-E1 preosteoblastic cells. Another study reported that exposure of mouse primary osteoblasts to low-dose radiation (<2 Gy) increased ALP activity, mineralization, and the expression of several osteogenic genes [65]. Hayashi et al., have assessed differentiation impacts of IR on cells with low and high sensitivity [66]. They compared the clonogenic and differentiation potential of induced pluripotent stem (iPS) cells with mouse hematopoietic stem/progenitor cells (HSCs) and found that iPS cells were less sensitive to radiation (the D_0_ values of HSPCs and iPS cells were 1.11 and 1.85, respectively), and expression levels of Nanog, Oct-4, Brachyury, Nestin, and Afp were decreased in dose-dependent manner, resulting in a delay in differentiation in both types of cells. Furthermore, skeletal stem cell stemness is restored by employing mitigator agents such as psoralen and melatonin, which mitigate radiation-induced bone damage by upregulating GSK-3β and NRF2 through Akt, resulting in osteogenesis in vitro and in vivo [67,68].

#### 2.2.2. NRF2 Enhances Adipogenesis

**Figure 3:**
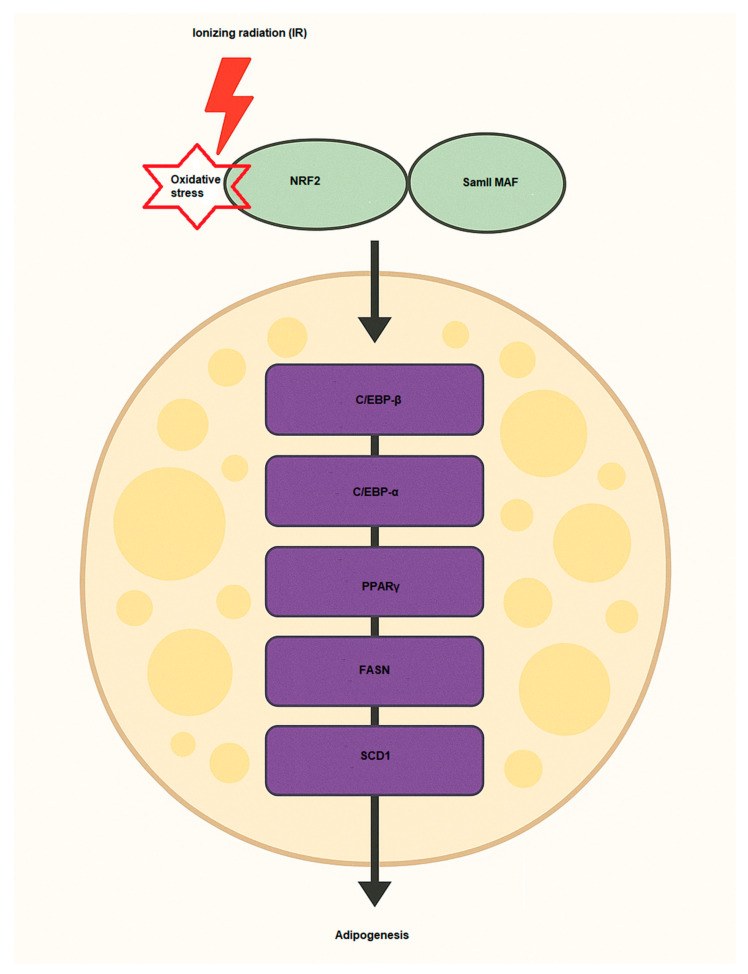
NRF2 activation induces adipogenesis via activation of key pro-adipogenic factors such as C/EBP-α, C/EBP-β, FASN, SCD1, and PPARγ. Samll MAF, musculoaponeurotic fibrosarcoma; SCD1, Stearoyl-CoA 9-desaturase; PPARγ, Peroxisome proliferator-activated receptor gamma; FASN, Fatty acid synthase; C/EBP, leucine zipper CCAAT-enhancer binding protein.

Multiple signaling pathways and proteins play crucial roles in driving the differentiation of preadipocytes into fully developed adipocytes. This process is regulated by the activation of essential transcription factors, including PPARγ (Peroxisome proliferator-activated receptor gamma), C/EBPα (CCAAT/enhancer-binding protein alpha), and SREBP-1c (Sterol regulatory element-binding protein 1c), which control lipid storage and the functional development of adipocytes [69,70]. The involvement of NRF2 in adipogenesis is significant, as it regulates various aspects of lipid metabolism, inflammation, and cellular oxidative stress in the development of adipose tissue [71] (Figure 3). NRF2 signaling has been shown to influence adipogenesis through several mechanisms [72]. One key pathway involves the regulation of transcription factors, where NRF2 can interact with adipogenic regulators such as PPARγ and enhance their expression. PPARγ is the master regulator of adipocyte differentiation and lipid storage. In order to promote adipocyte differentiation, NRF2 indirectly supports the activation of PPARγ and other adipogenic transcription factors by controlling oxidative stress levels [71]. Another effect is modulation of lipid metabolism. NRF2 influences lipid metabolism by regulating the expression of enzymes involved in fatty acid synthesis and storage. It can enhance lipid accumulation by promoting the expression of genes including FASN (fatty acid synthase) and SCD1 (Stearoyl-CoA desaturase 1), which are involved in the synthesis of fatty acids and the formation of triglycerides, key components of adipose tissue [73]. The other is via interaction with C/EBPα, which C/EBPα is another key transcription factor in adipogenesis. NRF2 can regulate the expression of C/EBPα, supporting the differentiation of preadipocytes into mature adipocytes [74,75]. NRF2 directly regulates the expression of Aryl hydrocarbon receptor (AHR), which inhibits adipocyte differentiation [76]. In Table 2, some studies regarding NRF2 effects on adipogenesis have been summarized. Under oxidative stress conditions, NRF2 expression and activity are induced, promoting lipid accumulation in adipocytes. This occurs through NRF2-mediated recruitment to the SREBP-1 promoter, enhancing lipogenesis and inhibition of lipolysis via the PKA pathway [77]. Consequently, NRF2 deficiency impairs adipogenesis, while its activation enhances adipocyte differentiation and lipid accumulation, highlighting its central role in adipocyte biology and obesity development. Recently, Yu and colleagues showed that IR promotes adipogenesis of MSCs in mice [78]. They used bioinformatics analysis (Oil Red O staining) and qPCR for the changes of adipogenic differentiation and oxidative stress pathways of MSCs and detected some markers such as *Cebpa*, *Lpl*, and *Pparg* after IR at level doses of 2 and 6 Gy. Their findings indicated that IR (2 Gy) enhanced MSC adipogenesis, with elevated expression of the genes NRF2 related to oxidative stress and Cebpa, Lpl, and Pparg. Sergeeva et al., have also assessed the effects of low doses of IR on human adipose-derived MSCs and found that 10 cGy of IR causes oxidized cell-free DNA (cfDNA) to be released and some of the cell population to die. Thus, cfDNA can enter the cytoplasm of neighbor cells and, similar to low doses of IR, cause oxidative stress and activation of antioxidant response (NRF2). These bystander effects lead to radioadaptive responses, inhibition of cell death, and proliferation of human adipose-derived MSCs [79]. Hammad et al., evaluated the LD50 of various kinds of IR, including X-rays, protons, and carbon ions, on human adipose-derived SCs after 8 days and observed that, according to their findings, when all applied radiation qualities are used, NRF2 inhibition decreases stem cell differentiation by 35% for adipogenesis and 28% for osteogenesis.

Remarkably, they showed that The surviving cells exposed to proton and carbon ion radiation are more capable of differentiating into the osteogenesis and adipogenesis lineages than cells exposed to X-ray radiation [1]. In addition to causing cutaneous lipid remodeling, electron radiation also protects the skin from damage caused by adipocytes via downregulating multiple pathways. Moreover, mature adipocytes encouraged the migration of co-cultured skin fibroblasts and keratinocytes, but not their proliferation. Furthermore, it is possible that fatty acid-binding protein 4 (FABP4) will be integrated into skin cells and help irradiated skin fibroblasts repair DNA damage [80]. It is reported that CRIF1 (cellular DNA-binding protein that interacts with the FGF-1) is a nuclear protein that plays a role in various cellular processes, including cell survival and stress response. CRIF1 is necessary for 3T3-L1 cell adipogenesis, which has been shown to have additional biological roles, such as regulating transcriptional activity through interactions with its DNA binding domain and promoting cell proliferation through interactions with CKII and CKBBP2/CRIF1 phosphorylation [81,82]. CRIF1 regulates NRF2 protein stability by proteasome-mediated degradation [83]. However, following IR exposure, CRIF1 stimulates the PKA/CREB signaling pathway to promote the adipogenic differentiation of bone marrow mesenchymal stem cells [84].

**Table 2:** IR effect on adipogenesis in different studies.

Source of IR or Oxidative Stress	Dose (Dose Rate)	Time After IR	Type of Stem Cells	Type of Study	Markers	Methods	Effect (Differentiation/Proliferation)	References
Cobalt-60	2 and 6 Gy (0.98 Gy/min)	-	MSCs	In vitro	Cebpa, Lpl, Pparg, and NRF2	Light microscope, RT-qPCR, and Western blotting	Adipogenesis	[78]
X-ray tube	10 cGy (10 cGy/min)	3–48 h	human adipose-derived MSCs	In vitro	NOX4 oxidase, NRF2	Flow Cytometry, fluorescence Microscopy, qRT-PCR, mass spectrometry, and DNA oxidation assay by UV spectrophotometry	Activation of DNA repair and cell proliferation	[79]
X-rays, proton and carbon ions	LD50 (2 Gy/min)	8–28 days	human adipose-derived MSCs	In vitro	HO-1, NQO1, and NRF2	Clonogenic, Western blotting, and cell prolifiration	Adipogenesis, osteogenesis, and cell proliferation	[1]
Cobalt-60	9 Gy (0.69 Gy/min)	24 h	Bone marrow-MSCs	In vitro	RUNX2, PPARγ, and CRIF1	Colony Formation, light microscope, RT-qPCR, Western blotting, and immunofluorescence	Adipogenesis and osteogenesis	[84]

#### 2.2.3. NRF2 Enhances Neurogenesis

**Figure 4:**
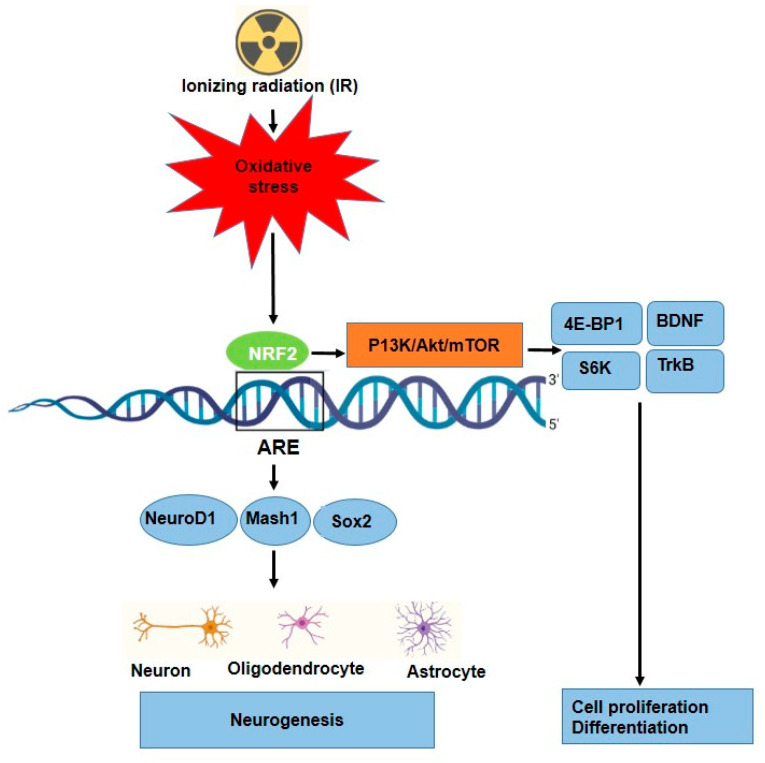
Proposed mechanism for neuronal differentiation-induced NRF2 signaling as well as P13K/Akt/mTOR signaling. NRF2 protein levels increase along with ARE activation.

Neurogenesis is tightly regulated by transcription factors such as NeuroD1, Mash1, and Sox2, which promote neuronal differentiation [85]. The role of NRF2 in neurogenesis has been reported in different studies, Table 3. NRF2 has been implicated in regulating the differentiation of neural progenitor cells into neurons and glial cells [86]. NRF2 is essential for both the induction of protective genes, such as HO-1, and injury-induced brain neurogenesis. For example, NRF2 signaling enhances NeuroD1 expression, which is crucial for the differentiation of NSCs into functional neurons, particularly in the hippocampus (region for learning and memory). However, NRF2 regulates NSCs proliferation, differentiation, and fate commitment during both embryonic and adult neurogenesis [48,87] (Figure 4). Additionally, it interacts with the mTOR pathway to control cellular growth, proliferation, and metabolism in neural development via some key factors such as 4E-BP1 and S6K [45,88]. Furthermore, NRF2 provides protection against amyloid-beta toxicity in neural progenitor cells, suggesting its potential therapeutic role in neurodegenerative diseases such as Alzheimer’s [86]. Huang et al., showed that through the stimulation of the NRF2-ARE signaling pathway, ADSC exosomes reduced oxidative stress of neuronal damage and lessened methotrexate (MTX)-induced neuronal damage [89]. Several studies have shown that oxidative stress can be a significant trigger for NRF2 activation in a variety of stem cells, such as HSCs [90], neuronal progenitor cells (NPCs) [86], and airway basal stem cells (ABSCs) [91], which leads to their proliferation and differentiation. Furthermore, role of IR as a source of oxidative stress to neurogenesis in some research has been investigated. Research has reported that irradiating neural precursor cells obtained from newborn mice at 8 Gy did not significantly alter neuronal differentiation [92], but 1 Gy of X-ray on NSCs prepared from mouse embryonic stem cells led to proliferation and differentiation [92]. Eom and colleagues have shown that IR (1–6 Gy) increased the expression of synaptophysin, synaptotagmin1, and GABA receptor mRNA in C17.2 stem cells, similar to normal differentiation by neurotrophin stimulation. Moreover, the findings show that IR can stimulate PI3K-STAT3-mGluR1 and PI3K-p53 signaling to cause altered neuronal development in undifferentiated neural stem-like cells [93]. Other signaling and molecular pathways affected in neurogenesis have been reported; for instance, Ramanan and colleagues findings point to a new function for PPARα ligands in enhancing neurogenesis after whole-brain irradiation at 10 Gy and hold out hope for enhancing the prognosis of patients with brain cancer undergoing radiation therapy [94]. Furthermore, it was observed that 0.3 Gy irradiation of mouse NSC cultures and the entire forebrain stimulated the Wnt/β-catenin signaling pathway, which controls NSC proliferation and differentiation as well as hippocampus neurogenesis [95].

The role of NRF2 to enhance neurogenesis after IR has been investigated by Liao et al. [96]. They investigated the role of sodium valproate (VPA) in a hippocampal neuronal cell line (HT22) and the hippocampus of zebrafish after exposure to IR. Data illustrated that VPA activated the NRF2/HO-1 pathway, expression of NRF2, and protein expression of HO-1 to prevent radiation-induced neuronal injury. It seems the role of NRF2 is a double-edged sword. Because, despite the fact that NRF2 is known to control the expression and coordinated induction of a number of cytoprotective genes that help protect cells and prevent inflammation, neoplasia, and neurodegeneration, it is also known that persistent accumulation or activation of NRF2 in the nucleus can promote oncogenesis and lead to drug resistance [34]. Based on our search and review, there is not enough research about the direct effect of IR on the activation of NRF2 signaling in neurogenesis.

## 3. Conclusions and Prospective View

IR, as an oxidative source, has a profound impact on cellular processes, including the activation of the NRF2 signaling pathway. NRF2 activation has been shown to modulate the balance between stem cell self-renewal and differentiation. IR, through the induction of oxidative stress, can activate NRF2 signaling, which may influence stem cell behavior. Understanding the dual roles of NRF2 in cellular protection and redox homeostasis offers a unique opportunity to tailor interventions in both oncology and regenerative medicine. In radiotherapy, leveraging NRF2 activation could enable the selective protection of normal tissue (particularly normal stem cell populations) without compromising the cytotoxic effects of IR on tumor cells [12,97]. Additionally, controlled exposure to low-dose IR as a preconditioning strategy may have translational relevance for improving the viability, engraftment, and therapeutic outcomes of stem cell-based therapies for degenerative diseases. A study found that NRF2 levels and serum antioxidant capacity increased after 5 × 2 Gy of radiotherapy, with stronger antioxidant responses associated with poorer local tumor control, highlighting the need to carefully balance NRF2 activation [98]. By translating findings from animal models and in vitro studies into clinically viable interventions, this line of research paves the way for novel treatment paradigms that minimize side effects while enhancing tissue regeneration and functional recovery.

The effects of IR on stem cell differentiation are complex and can vary depending on the dose radiation quality and timing of radiation exposure. At moderate doses, IR may promote differentiation by enhancing NRF2-mediated protection against oxidative damage. However, excessive or chronic exposure to IR may lead to detrimental effects such as DNA damage, cellular senescence, and tumorigenesis of cells. The activation of NRF2 in this context may serve as a protective mechanism, but it could also contribute to a pro-survival response that supports the persistence of abnormal or damaged cells. However, the relationship between IR and NRF2 signaling in stem cell differentiation is an area that requires further exploration. Future research should focus on unraveling the molecular mechanisms underlying NRF2 activation following IR exposure. This would help define how stem cells respond to oxidative stress and how NRF2 modulates differentiation pathways. In summary, despite the fact that the role of NRF2 in response to oxidative stress of IR has been proven, research in this field appears to be primary. Still some challenges remain to be solved. For instance, there is not an agreement regarding the dose and dose rate for which differentiation is induced. Nonetheless, some studies indicated that differentiation occurred at doses below 2 Gy, while others observed a single treatment exceeding 2 Gy. Understanding the threshold dose at which NRF2 activation is beneficial or harmful could inform safe radiation exposure protocols. Another key factor that needs to be considered is bystander effects of damaged and undamaged cells, which can affect cell proliferation and differentiation. Moreover, response time after IR exposure for various stem cells is different. Herby, some research mentioned a short time (24 h) after treatment, while others reported a long time (days and weeks). Another important feature to consider was the sensitivity of various stem cells. For example, activation of NRF2 in neurogenesis can occur at high doses, but for other sensitive stem cells, it happens at low doses. For instance, high dosages of IR lead to NRF2 activation in neurogenesis, but modest amounts are enough to activate NRF2 in other sensitive stem cells. As mentioned in the text, in response to oxidative stress, NRF2 has crosstalk with other signaling pathways such as PI3K-p53, Akt signaling, and Wnt/β-catenin that could affect differentiation efficiency. However, consideration of this point for the next investigation is important. The next recommendation for the future is using NFR2 activators or some mitigator drugs such as psoralen, curcumin, or resveratrol to alleviate radiation-induced injury and could have a positive effect on cell differentiation synergistically.

Finally, despite promising findings, significant gaps remain in our understanding of how IR enhances the proliferation and differentiation of normal stem cells via NRF2 activation. Much of the current evidence is derived from in vitro studies using immortalized cell lines, which may not accurately represent the behavior of primary human stem cells. Moreover, in vivo studies, especially those involving human-relevant models, are limited, making it difficult to assess the physiological relevance and therapeutic potential of NRF2-mediated effects in complex tissue environments. Additionally, dose optimization, timing, and tissue-specific responses to IR remain poorly defined. Addressing these gaps will require rigorous in vivo experimentation and validation using primary human stem cells to support safe and effective clinical translation.

## Figures and Tables

**Table 1:** IR effect on osteogenesis in various studies.

Source of IR or Oxidative Stress	Dose (Dose Rate)	Time After IR	Type of Stem Cells	Type of Study	Evaluated Markers	Methods	Effect (Differentiation/Proliferation)	**References**
^137^Cs	1.125 (not mentioned)	48 h	HSCs	In vitro	SDF-1 (CXCL12)	Radiolabling for proliferating cells, immunohistochemistry, microscopy I mage, and qPCR	Enhanced osteogenesis	[61]
^137^Cs	6.9–7.3 (0.53 Gy/min)	1–21 days	HSCs	In vivo	KEAP1, KI67 markers, and JAG1	qPCR, flow cytometry and colony formation	Enhanced osteogenesis	[13]
t-BHQ (Solarbio)	1, 5, 10 μM	36 h	Periodontal ligament stem cells (PDLSCs)	In vitro and in vivo	HO-1, NQO-1, and RUNX2	PDLSC isolation, flow cytometry, qPCR, western blotting, proteomics, micro-CT, and mineralization nodules via Alizarin Red	Enhanced osteogenesis	[62]
Cobalt-60	250 m Gy-2.5 Gy (100 mGy/min and 1 Gy/min, respectively)	6–8 days (in vitro) and 12–14 days (in vivo)	HSPCs	In vitro and in vivo	Thr180/Tyr18	Immunomagnetic, flow cytometry, and Western blotting	Proliferation	[63]
A linear accelerator (X-ray)	<2 Gy (1.5 Gy/min)	3–7 days	Primary osteoblasts	In vivo	ALP, collagen I, osteopontin, and osteocalcin	Alkaline Phosphatase (ALP) activity, Alizarin Red S staining, RT-PCR, and Western blotting	Enhanced osteogenesis	[65]
X-rays	1, 2, 4, or 7.5 Gy (3.3–3.4 Gy/min)	6 days	Induced pluripotent stem (iPS) and HSPCs	In vitro/In vivo	Afp, Nanog, and Oct-4	Clonogenic assay, Embryoid Body (EB) formation assay, and gene expression	Delay in differentiation	[66]
Cobalt-60	2 Gy (0.98 Gy/min)	1–7 days	Skeletal stem cells (SSCs)	In vitro/In vivo	p-Akt, NRF2, KEAP1, GSK-3β, p-GSK-3β, HMOX1, and NQO1	Micro-CT, proliferation assay, colony formation, qPCR, and Western blotting	Regenerative, proliferation, and osteogenic differentiation	[67]

**Table 3:** IR enhances neurogenesis.

Source of IR or Oxidative Stress	Dose (Dose Rate)	Time After IR or Oxidative Stress	Type of Stem Cells	Type of Study	Markers	Methods	Effect (Differentiation/Proliferation)	References
5-FU chemotherapy	-	5–10 days	Primary hematopoietic cells	In vitro/In vivo	Granulocyte/erythroid markers, NRF2, and KEAP1	Flow cytometry and qRT-PCR	KEAP1–NRF2 axis is a critical regulator of HSC fate and healthy hematopoiesis	[90]
γ-ray (^137^Cs source)	0–8 Gy (note reported)	24–48 h	NSPs	In vitro/In vivo	Nestin. NRF2, Sox2, GFAP, NG2, and neurospheres	Neurosphere formation, immunocytochemistry, cell-cycle flow cytometry, and Western blotting	Ability to generate neurons, astrocytes, and oligodendrocytes post-irradiation	[92]
γ-ray	2–6 Gy (not reported)	72 h	NSCs	In vitro/In vivo	Nestin, β-III Tubulin mGluR1, phospho-Akt, and p53	Immunofluorescence, Western blotting, and qPCR	Radiation triggers neuronal differentiation	[93]
γ-ray (^137^Cs source)	Single acute dose of 10 Gy (3.33 Gy/min)	1 week and 2 months post-irradiation	NPCs	In vitro/In vivo	BrdU/NeuN, Ki-67, CD68, and PPARα	Immunofluorescence, immunohistochemistry, and CD68 staining	Fenofibrate preserves survival	[94]
γ-ray (^137^Cs source)	0.3–3 Gy (not reported)	2 days-4 weeks	NSCs	In vitro/In vivo	Nestin, Wnt1, Wnt3a, Wnt5a, and β-catenin	Flow cytometry, immunohistochemistry, and RT-PCR	Enhanced cell survival, stem cell proliferation and neurogenesis	[95]
X-ray (6-MV linear accelerator)	6 Gy and 20 Gy (5.0 Gy/min)	24 h	HT22 cells	In vitro/In vivo	NRF2, HO-1, NeuN, and MAP2	Immunohistochemistry and Western blotting	Neuroprotective effects	[96]

## Data Availability

Not applicable.

## References

[1] Hammad M., Salma R., Balosso J., Rezvani M., Haghdoost S. (2024). Role of oxidative stress signaling, Nrf2, on survival and stemness of human adipose-derived stem cells exposed to X-rays, protons and carbon ions. Antioxidants.

[2] Waheed T.O., Hahn O., Sridharan K., Mörke C., Kamp G., Peters K. (2022). Oxidative stress response in adipose tissue-derived mesenchymal stem/stromal cells. Int. J. Mol. Sci..

[3] Li Y., Yue G., Yu S., Liu Z., Cao Y., Wang X. (2024). Extracellular Vesicles Derived from H2O2-Stimulated Adipose-Derived Stem Cells Alleviate Senescence in Diabetic Bone Marrow Mesenchymal Stem Cells and Restore Their Osteogenic Capacity. Drug Des. Dev. Ther..

[4] Carrière A., Ebrahimian T.G., Dehez S., Augeé N., Joffre C., Andreé M., Arnal S., Duriez M., Barreau C., Arnaud E. (2009). Preconditioning by mitochondrial reactive oxygen species improves the proangiogenic potential of adipose-derived cells-based therapy. Arterioscler. Thromb. Vasc. Biol..

[5] Goudarzi F., Mohammadalipour A., Bahabadi M., Goodarzi M.T., Sarveazad A., Khodadadi I. (2018). Hydrogen peroxide: A potent inducer of differentiation of human adipose-derived stem cells into chondrocytes. Free Radic. Res..

[6] Rochette L., Mazini L., Malka G., Zeller M., Cottin Y., Vergely C. (2020). The crosstalk of adipose-derived stem cells (ADSC), oxidative stress, and inflammation in protective and adaptive responses. Int. J. Mol. Sci..

[7] Ghosh S., Ghosh A. (2021). Activation of DNA damage response signaling in mammalian cells by ionizing radiation. Free Radic. Res..

[8] Liu Y., Jiang B., Li Y., Zhang X., Wang L., Yao Y., Zhu B., Shi H., Chai X., Hu X. (2024). Effect of traditional Chinese medicine in osteosarcoma: Cross-interference of signaling pathways and potential therapeutic targets. Medicine.

[9] Zhan M., Han Z.C. (2004). Phosphatidylinositide 3-kinase AKT in radiation responses. Histol. Histopathol..

[10] Serrano Martinez P., Giuranno L., Vooijs M., Coppes R.P. (2021). The radiation-induced regenerative response of adult tissue-specific stem cells: Models and signaling pathways. Cancers.

[11] Sekhar K.R., Freeman M.L. (2015). Nrf2 promotes survival following exposure to ionizing radiation. Free Radic. Biol. Med..

[12] Chute J.P. (2014). NRF2 mitigates radiation-induced hematopoietic death. J. Clin. Investig..

[13] Kim J.-H., Thimmulappa R.K., Kumar V., Cui W., Kumar S., Kombairaju P., Zhang H., Margolick J., Matsui W., Macvittie T. (2014). NRF2-mediated Notch pathway activation enhances hematopoietic reconstitution following myelosuppressive radiation. J. Clin. Investig..

[14] Li J., Xu C., Liu Q. (2023). Roles of NRF2 in DNA damage repair. Cell. Oncol..

[15] Xu N., Lao Y., Zhang Y., Gillespie D.A. (2012). Akt: A double-edged sword in cell proliferation and genome stability. J. Oncol..

[16] Marwarha G., Ghribi O. (2017). Nuclear Factor Kappa-light-chain-enhancer of Activated B Cells (NF-ΚB)–a Friend, a Foe, or a Bystander-in the Neurodegenerative Cascade and Pathogenesis of Alzheimer’s Disease. CNS Neurol. Disord.-Drug Targets (Former. Curr. Drug Targets-CNS Neurol. Disord.).

[17] Singh V., Gupta D., Arora R. (2015). NF-kB as a key player in regulation of cellular radiation responses and identification of radiation countermeasures. Discoveries.

[18] Oeckinghaus A., Hayden M.S., Ghosh S. (2011). Crosstalk in NF-κB signaling pathways. Nat. Immunol..

[19] McDonald J.T., Kim K., Norris A.J., Vlashi E., Phillips T.M., Lagadec C., Della Donna L., Ratikan J., Szelag H., Hlatky L. (2010). Ionizing radiation activates the Nrf2 antioxidant response. Cancer Res..

[20] Baeyens A., Abrantes A.M., Ahire V., Ainsbury E.A., Baatout S., Baselet B., Botelho M.F., Boterberg T., Chevalier F., Da Pieve F. (2023). Basic concepts of radiation biology. Radiobiology Textbook.

[21] Akbari A., Jelodar G., Nazifi S., Afsar T., Nasiri K. (2019). Oxidative stress as the underlying biomechanism of detrimental outcomes of ionizing and non-ionizing radiation on human health: Antioxidant protective strategies. Zahedan J. Res. Med. Sci..

[22] Kuzmić M. (2018). Role of Protein and DNA Damage in Biological Response to Radiation and Aging. Ph.D. Thesis.

[23] Moloudi K., Neshasteriz A., Hosseini A., Eyvazzadeh N., Shomali M., Eynali S., Mirzaei E., Azarnezhad A. (2017). Synergistic effects of arsenic trioxide and radiation: Triggering the intrinsic pathway of apoptosis. Iran. Biomed. J..

[24] Sangsuwan T., Khavari A.P., Blomberg E., Romell T., De Godoy P.R.d.V., Harms-Ringdahl M., Haghdoost S. (2023). Oxidative stress levels and dna repair kinetics in senescent primary human fibroblasts exposed to chronic low dose rate of ionizing radiation. Front. Biosci.-Landmark.

[25] Nuszkiewicz J., Woźniak A., Szewczyk-Golec K. (2020). Ionizing radiation as a source of oxidative stress—The protective role of melatonin and vitamin D. Int. J. Mol. Sci..

[26] Sampadi B., Mullenders L.H., Vrieling H. (2022). Low and high doses of ionizing radiation evoke discrete global (phospho) proteome responses. DNA Repair.

[27] Dettmering T., Zahnreich S., Colindres-Rojas M., Durante M., Taucher-Scholz G., Fournier C. (2015). Increased effectiveness of carbon ions in the production of reactive oxygen species in normal human fibroblasts. J. Radiat. Res..

[28] Merchant A.A., Singh A., Matsui W., Biswal S. (2011). The redox-sensitive transcription factor Nrf2 regulates murine hematopoietic stem cell survival independently of ROS levels. Blood J. Am. Soc. Hematol..

[29] Konkova M., Abramova M., Kalianov A., Ershova E., Dolgikh O., Umriukhin P., Izhevskaya V., Kutsev S., Veiko N., Kostyuk S. (2020). Mesenchymal stem cells early response to low-dose ionizing radiation. Front. Cell Dev. Biol..

[30] Rodrigues-Moreira S., Moreno S.G., Ghinatti G., Lewandowski D., Hoffschir F., Ferri F., Gallouet A.-S., Gay D., Motohashi H., Yamamoto M. (2017). Low-dose irradiation promotes persistent oxidative stress and decreases self-renewal in hematopoietic stem cells. Cell Rep..

[31] Yamamoto M., Kensler T.W., Motohashi H. (2018). The KEAP1-NRF2 system: A thiol-based sensor-effector apparatus for maintaining redox homeostasis. Physiol. Rev..

[32] Murakami S., Motohashi H. (2015). Roles of Nrf2 in cell proliferation and differentiation. Free Radic. Biol. Med..

[33] Jang J., Wang Y., Kim H.-S., Lalli M.A., Kosik K.S. (2014). Nrf2, a regulator of the proteasome, controls self-renewal and pluripotency in human embryonic stem cells. Stem Cells.

[34] Kaspar J.W., Niture S.K., Jaiswal A.K. (2009). Nrf2: INrf2 (Keap1) signaling in oxidative stress. Free Radic. Biol. Med..

[35] Suzuki T., Muramatsu A., Saito R., Iso T., Shibata T., Kuwata K., Kawaguchi S.-i., Iwawaki T., Adachi S., Suda H. (2019). Molecular mechanism of cellular oxidative stress sensing by Keap1. Cell Rep..

[36] Sajadimajd S., Khazaei M. (2018). Oxidative stress and cancer: The role of Nrf2. Curr. Cancer Drug Targets.

[37] Maruyama A., Itoh K. (2015). Role of Keap1/Nrf2 pathway in the protection against ionizing radiation. Fukushima Nuclear Accident: Global Implications, Long-Term Health Effects and Ecological Consequences.

[38] Sun X., Wang Y., Ji K., Liu Y., Kong Y., Nie S., Li N., Hao J., Xie Y., Xu C. (2020). NRF2 preserves genomic integrity by facilitating ATR activation and G2 cell cycle arrest. Nucleic Acids Res..

[39] Jayakumar S., Pal D., Sandur S.K. (2015). Nrf2 facilitates repair of radiation induced DNA damage through homologous recombination repair pathway in a ROS independent manner in cancer cells. Mutat. Res./Fundam. Mol. Mech. Mutagen..

[40] Zhong C.-C., Zhao T., Hogstrand C., Chen F., Song C.-C., Luo Z. (2022). Copper (Cu) induced changes of lipid metabolism through oxidative stress-mediated autophagy and Nrf2/PPARγ pathways. J. Nutr. Biochem..

[41] Taha R., Blaise G. (2014). Nrf2 activation as a future target of therapy for chronic diseases. Funct. Foods Health Dis..

[42] Evans J.A., Mendonca P., Soliman K.F. (2023). Involvement of Nrf2 activation and NF-kB pathway inhibition in the antioxidant and anti-inflammatory effects of hesperetin in activated BV-2 microglial cells. Brain Sci..

[43] Wang L., He C. (2022). Nrf2-mediated anti-inflammatory polarization of macrophages as therapeutic targets for osteoarthritis. Front. Immunol..

[44] Mukherjee R., Rana R., Mehan S., Khan Z., Das Gupta G., Narula A.S., Samant R. (2025). Investigating the Interplay Between the Nrf2/Keap1/HO-1/SIRT-1 Pathway and the p75NTR/PI3K/Akt/MAPK Cascade in Neurological Disorders: Mechanistic Insights and Therapeutic Innovations. Mol. Neurobiol..

[45] Zoungrana L.I., Krause-Hauch M., Wang H., Fatmi M.K., Bates L., Li Z., Kulkarni P., Ren D., Li J. (2022). The interaction of mTOR and Nrf2 in neurogenesis and its implication in neurodegenerative diseases. Cells.

[46] Xu C., Wang J., Zhu T., Shen Y., Tang X., Fang L., Xu Y. (2016). Cross-talking between PPAR and WNT signaling and its regulation in mesenchymal stem cell differentiation. Curr. Stem Cell Res. Ther..

[47] Yuan Z., Li Q., Luo S., Liu Z., Luo D., Zhang B., Zhang D., Rao P., Xiao J. (2016). PPARγ and Wnt signaling in adipogenic and osteogenic differentiation of mesenchymal stem cells. Curr. Stem Cell Res. Ther..

[48] Boorman E., Killick R., Aarsland D., Zunszain P., Mann G.E. (2022). NRF2: An emerging role in neural stem cell regulation and neurogenesis. Free Radic. Biol. Med..

[49] Hannan M.A., Dash R., Sohag A.A.M., Haque M.N., Moon I.S. (2020). Neuroprotection against oxidative stress: Phytochemicals targeting TrkB signaling and the Nrf2-ARE antioxidant system. Front. Mol. Neurosci..

[50] Sheppard A.J., Barfield A.M., Barton S., Dong Y. (2022). Understanding reactive oxygen species in bone regeneration: A glance at potential therapeutics and bioengineering applications. Front. Bioeng. Biotechnol..

[51] Onoki T., Kanczler J., Rawlings A., Smith M., Kim Y.H., Hashimoto K., Aizawa T., Oreffo R.O. (2024). Modulation of osteoblastogenesis by NRF2: NRF2 activation suppresses osteogenic differentiation and enhances mineralization in human bone marrow-derived mesenchymal stromal cells. FASEB J..

[52] Park C.K., Lee Y., Kim K.H., Lee Z.H., Joo M., Kim H.-H. (2014). Nrf2 is a novel regulator of bone acquisition. Bone.

[53] Han J., Yang K., An J., Jiang N., Fu S., Tang X. (2022). The role of NRF2 in bone metabolism–Friend or foe?. Front. Endocrinol..

[54] Gao Y., Huang E., Zhang H., Wang J., Wu N., Chen X., Wang N., Wen S., Nan G., Deng F. (2013). Crosstalk between Wnt/β-catenin and estrogen receptor signaling synergistically promotes osteogenic differentiation of mesenchymal progenitor cells. PLoS ONE.

[55] Rana T., Schultz M.A., Freeman M.L., Biswas S. (2012). Loss of Nrf2 accelerates ionizing radiation-induced bone loss by upregulating RANKL. Free Radic. Biol. Med..

[56] Barcellos-Hoff M. (1993). Radiation-induced transforming growth factor β and subsequent extracellular matrix reorganization in murine mammary gland. Cancer Res..

[57] Cameron B.D., Sekhar K.R., Ofori M., Freeman M.L. (2018). The role of Nrf2 in the response to normal tissue radiation injury. Radiat. Res..

[58] Ambrożewicz E., Tokajuk G., Muszyńska M., Zaręba I., Skrzydlewska E. (2019). Cross talk between redox signalling and metabolic activity of osteoblasts and fibroblasts in the presence of hydroxyapatite-based biomaterials influences bone regeneration. J. Appl. Biomed..

[59] Bohen S., O’Connor M.J., Morgan M. (2018). DNA Damage Response–An Emerging Target for Groundbreaking Cancer Therapies. J.-DNA Damage Response–Emerg. Target Groundbreaking Cancer Ther..

[60] Oest M.E., Franken V., Kuchera T., Strauss J., Damron T.A. (2015). Long-term loss of osteoclasts and unopposed cortical mineral apposition following limited field irradiation. J. Orthop. Res..

[61] Dominici M., Rasini V., Bussolari R., Chen X., Hofmann T.J., Spano C., Bernabei D., Veronesi E., Bertoni F., Paolucci P. (2009). Restoration and reversible expansion of the osteoblastic hematopoietic stem cell niche after marrow radioablation. Blood J. Am. Soc. Hematol..

[62] Xi X., Zhao Y., Liu H., Li Z., Chen S., Liu D. (2021). Nrf2 activation is involved in osteogenic differentiation of periodontal ligament stem cells under cyclic mechanical stretch. Exp. Cell Res..

[63] Henry E., Souissi-Sahraoui I., Deynoux M., Lefèvre A., Barroca V., Campalans A., Ménard V., Calvo J., Pflumio F., Arcangeli M.-L. (2019). Human hematopoietic stem/progenitor cells display ROS-dependent long-term hematopoietic defects after exposure to low dose of ionizing radiations. Haematologica.

[64] Kook S.-H., Kim K.-A., Ji H., Lee D., Lee J.-C. (2015). Irradiation inhibits the maturation and mineralization of osteoblasts via the activation of Nrf2/HO-1 pathway. Mol. Cell. Biochem..

[65] Park S.-S., Kim K.-A., Lee S.-Y., Lim S.-S., Jeon Y.-M., Lee J.-C. (2012). X-ray radiation at low doses stimulates differentiation and mineralization of mouse calvarial osteoblasts. BMB Rep..

[66] Hayashi N., Monzen S., Ito K., Fujioka T., Nakamura Y., Kashiwakura I. (2012). Effects of ionizing radiation on proliferation and differentiation of mouse induced pluripotent stem cells. J. Radiat. Res..

[67] Yin B.-F., Li Z.-L., Yan Z.-Q., Guo Z., Liang J.-W., Wang Q., Zhao Z.-D., Li P.-L., Hao R.-C., Han M.-Y. (2022). Psoralen alleviates radiation-induced bone injury by rescuing skeletal stem cell stemness through AKT-mediated upregulation of GSK-3β and NRF2. Stem Cell Res. Ther..

[68] Hu W., Liang J.-W., Liao S., Zhao Z.-D., Wang Y.-X., Mao X.-F., Hao S.-W., Wang Y.-F., Zhu H., Guo B. (2021). Melatonin attenuates radiation-induced cortical bone-derived stem cells injury and enhances bone repair in postradiation femoral defect model. Mil. Med. Res..

[69] Moseti D., Regassa A., Kim W.-K. (2016). Molecular regulation of adipogenesis and potential anti-adipogenic bioactive molecules. Int. J. Mol. Sci..

[70] Tang Q.Q., Lane M.D. (2012). Adipogenesis: From stem cell to adipocyte. Annu. Rev. Biochem..

[71] Wang Z., Zuo Z., Li L., Ren S., Gao T., Fu J., Hou Y., Chen Y., Pi J. (2020). Nrf2 in adipocytes. Arch. Pharmacal Res..

[72] Seo H.-A., Lee I.-K. (2013). The role of Nrf2: Adipocyte differentiation, obesity, and insulin resistance. Oxidative Med. Cell. Longev..

[73] Lauren Tebay B. (2015). Investigating the Role of Transcription Factors Nrf2 and Pparα in Hepatic Lipid Metabolism During Fasting. Ph.D. Thesis.

[74] Pi J., Leung L., Xue P., Wang W., Hou Y., Liu D., Yehuda-Shnaidman E., Lee C., Lau J., Kurtz T.W. (2010). Deficiency in the nuclear factor E2-related factor-2 transcription factor results in impaired adipogenesis and protects against diet-induced obesity. J. Biol. Chem..

[75] Hou Y., Xue P., Bai Y., Liu D., Woods C.G., Yarborough K., Fu J., Zhang Q., Sun G., Collins S. (2012). Nuclear factor erythroid-derived factor 2-related factor 2 regulates transcription of CCAAT/enhancer-binding protein β during adipogenesis. Free Radic. Biol. Med..

[76] Shin S., Wakabayashi N., Misra V., Biswal S., Lee G.H., Agoston E.S., Yamamoto M., Kensler T.W. (2007). NRF2 modulates aryl hydrocarbon receptor signaling: Influence on adipogenesis. Mol. Cell. Biol..

[77] Gu W., Wu G., Chen G., Meng X., Xie Z., Cai S. (2024). Polyphenols alleviate metabolic disorders: The role of ubiquitin-proteasome system. Front. Nutr..

[78] Yu F.-H., Yin B.-F., Li P.-L., Li X.-T., Tian J.-Y., Xu R.-X., Tang J., Zhang X.-Y., Zhang W.-J., Zhu H. (2025). The Enhancing Effects and Underlying Mechanism of Ionizing Radiation on Adipogenic Differentiation of Mesenchymal Stem Cells via Regulating Oxidative Stress Pathway. Zhongguo Shi Yan Xue Ye Xue Za Zhi.

[79] Sergeeva V., Ershova E., Veiko N., Malinovskaya E., Kalyanov A., Kameneva L., Stukalov S., Dolgikh O., Konkova M., Ermakov A. (2017). Low-Dose Ionizing Radiation Affects Mesenchymal Stem Cells via Extracellular Oxidized Cell-Free DNA: A Possible Mediator of Bystander Effect and Adaptive Response. Oxidative Med. Cell. Longev..

[80] Xiao Y., Mo W., Jia H., Yu D., Qiu Y., Jiao Y., Zhu W., Koide H., Cao J., Zhang S. (2020). Ionizing radiation induces cutaneous lipid remolding and skin adipocytes confer protection against radiation-induced skin injury. J. Dermatol. Sci..

[81] Kwon M.-c., Koo B.-K., Kim Y.-Y., Lee S.-H., Kim N.-S., Kim J.-H., Kong Y.-Y. (2009). Essential role of CR6-interacting factor 1 (Crif1) in E74-like factor 3 (ELF3)-mediated intestinal development. J. Biol. Chem..

[82] Oh N.-S., Yoon S.-H., Lee W.-K., Choi J.-Y., Min D.S., Bae Y.-S. (2007). Phosphorylation of CKBBP2/CRIF1 by protein kinase CKII promotes cell proliferation. Gene.

[83] Kang H.J., Hong Y.B., Kim H.J., Bae I. (2010). CR6-interacting factor 1 (CRIF1) regulates NF-E2-related factor 2 (NRF2) protein stability by proteasome-mediated degradation. J. Biol. Chem..

[84] Zhang X., Xiang L., Ran Q., Liu Y., Xiang Y., Xiao Y., Chen L., Li F., Zhong J.F., Li Z. (2015). Crif1 promotes adipogenic differentiation of bone marrow mesenchymal stem cells after irradiation by modulating the PKA/CREB signaling pathway. Stem Cells.

[85] Hsieh J. (2012). Orchestrating transcriptional control of adult neurogenesis. Genes Dev..

[86] Kärkkäinen V., Pomeshchik Y., Savchenko E., Dhungana H., Kurronen A., Lehtonen S., Naumenko N., Tavi P., Levonen A.-L., Yamamoto M. (2014). Nrf2 regulates neurogenesis and protects neural progenitor cells against Aβ toxicity. Stem Cells.

[87] Robledinos-Antón N., Rojo A.I., Ferreiro E., Núñez Á., Krause K.-H., Jaquet V., Cuadrado A. (2017). Transcription factor NRF2 controls the fate of neural stem cells in the subgranular zone of the hippocampus. Redox Biol..

[88] Bendavit G., Aboulkassim T., Hilmi K., Shah S., Batist G. (2016). Nrf2 transcription factor can directly regulate mTOR: Linking cytoprotective gene expression to a major metabolic regulator that generates redox activity. J. Biol. Chem..

[89] Huang T., Tong H., Zhou H., Wang J., Hu L., Wang Y., Huang Z. (2022). ADSC-exosomes alleviate MTX-induced rat neuronal damage by activating Nrf2-ARE pathway. J. Mol. Neurosci..

[90] Murakami S., Shimizu R., Romeo P.H., Yamamoto M., Motohashi H. (2014). Keap1-Nrf2 system regulates cell fate determination of hematopoietic stem cells. Genes Cells.

[91] Paul M.K., Bisht B., Darmawan D.O., Chiou R., Ha V.L., Wallace W.D., Chon A.T., Hegab A.E., Grogan T., Elashoff D.A. (2014). Dynamic changes in intracellular ROS levels regulate airway basal stem cell homeostasis through Nrf2-dependent Notch signaling. Cell Stem Cell.

[92] Chen H., Levison S., De Toledo S., Azzam E., Souayah N. (2012). Effects of Ionizing Radiation on Neural Precursor Cells (IN8-1.008).

[93] Eom H.S., Park H.R., Jo S.K., Kim Y.S., Moon C., Kim S.-H., Jung U. (2016). Ionizing radiation induces altered neuronal differentiation by mGluR1 through PI3K-STAT3 signaling in C17. 2 mouse neural stem-like cells. PLoS ONE.

[94] Ramanan S., Kooshki M., Zhao W., Hsu F.-C., Riddle D.R., Robbins M.E. (2009). The PPARα agonist fenofibrate preserves hippocampal neurogenesis and inhibits microglial activation after whole-brain irradiation. Int. J. Radiat. Oncol. * Biol. * Phys..

[95] Wei L.-C., Ding Y.-X., Liu Y.-H., Duan L., Bai Y., Shi M., Chen L.-W. (2012). Low-dose radiation stimulates Wnt/β-catenin signaling, neural stem cell proliferation and neurogenesis of the mouse hippocampus in vitro and in vivo. Curr. Alzheimer Res..

[96] Liao G., Li R., Chen X., Zhang W., Du S., Yuan Y. (2016). Sodium valproate prevents radiation-induced injury in hippocampal neurons via activation of the Nrf2/HO-1 pathway. Neuroscience.

[97] Panieri E., Buha A., Telkoparan-Akillilar P., Cevik D., Kouretas D., Veskoukis A., Skaperda Z., Tsatsakis A., Wallace D., Suzen S. (2020). Potential applications of NRF2 modulators in cancer therapy. Antioxidants.

[98] Reinema F., Kaanders J., Peeters W., Adema G., Sweep F., Bussink J., Span P. (2024). Radiotherapy induces an increase in serum antioxidant capacity reflecting tumor response. Clin. Transl. Radiat. Oncol..

